# 

**Published:** 1915-10-16

**Authors:** 


### PASSING TOPICS OF THE DAY.

### Red Cross Sisters Murdered by Germans.

According to the Petrograd correspondent of the *Petit Journal* the Russian Red Cross has published a list of forty-six Sisters of Charity who have perished as the result of the cannonade directed by the Austro-Germans on the Red Cross. A nation which deliberately slaughters Sisters of Charity when acting as nurses under the Red Cross at the Front, requires to be wiped out for the protection of the human race.

### Are Telephone Mouthpieces Harmless?

In the days before the war, when the newspaper Press could find an abundance of space for all sorts of quasi-medical topics, the public used to delight in scares. One of the contrivances we were told to avoid if we wished to escape from infectious diseases was the telephone mouthpiece. Quite recently we have had an echo of this rumour. We learn from the *Yorkshire Observer* that the West Riding Insurance Committee have been in communication with the Postmaster-General upon the question of whether mouthpieces of telephones can be a medium of infection. The reply received shows that the Department has given careful attention to the matter. As far back as ten years ago, bacteriological experiments were carried out in connection with public call office telephones. The result confirmed investigations previously made by Dr. Collingridge, medical officer for the City of London. In 1910 a further inquiry was caused to be made by Mr. Herbert Samuel, and telephone mouthpieces used by consumptives at two sanatoria were also examined. It was shown that the ordinary methods of cleaning telephones were quite efficacious, and the mouthpieces examined were found to be free from tubercle and diphtheria germs, the conclusion reached being that the transmission of tuberculosis through the medium of the telephone was practically impossible.

### Wounded Needs in Alexandria.

The *Westminster Gazette* has given a most interesting account of the many needs of the sufferers in No. 21 General Hospital, Alexandria, which is full of men from the Dardanelles and the Near East. There is great need for books, magazines, fly papers, and cheap fans; also for walking sticks, writing materials of all kinds, tobacco, cigarettes, cigarette papers, and pipes. There is a great wastage of air cushions and water beds owing to the necessity of burning those used by typhoid patients and others suffering from infectious disease. Cushions, extra feather pillows, pillow cases, old linen bandages of all sizes and kinds, and also muslin squares of varying sizes, weighted with beads, to cover cups and bowls, are in great demand. Oranges are not obtainable in Egypt at this season of the year, and boxes of oranges, for which the serious cases crave, would be a great advantage to many of the patients. Those patients who are sent home in hospital ships, or whose recovery enables them to return to the Front, are sadly in want of boxes of various sizes in which to pack their small possessions. Clothing of any kind, either thick or thin (the latter being required for convalescent patients on their way home), would prove a great boon. Many of the men, it is declared, love needlework, so materials for wool work, canvas, wools of all colours, needles, and small frames for making Daisy mats may be sent; and packs of cards, draught-boards, and so on are asked for. Anyone wishing to fill a case with any of the above things may send it direct, addressed to Mrs. Walrond, care of Lieut.-Colonel Oliver Robinson, Commandant, 21 General Hospital, Alexandria. Smaller consignments may be sent to Mrs. Walrond, 13 Gerald Road, London, S.W., when they will be sent forward by Lady Henry, the wife of the Chief Commissioner of the Metropolitan Police. Gifts of money should be addressed to Lady Henry, 29 Campden House Court, Sheffield Terrace, Kensington, W.

### The Norfolk and Norwich Hospital.

ITS WORK AND FINANCES IN WAR-TIME.

At the Quarterly Meeting of the Governors held on the 9th inst., the Chairman of the Board (Mr. C. S. Tomes) referred at length to the approaching departure of Mr. F. G. Hazell, who has recently been appointed superintendent and secretary to the Manchester Royal Infirmary. He stated in his remarks that during the fifteen years of Mr. Hazell's services in the Norfolk and Norwich Hospital, he had discharged the multifarious duties of his office in a manner which would render it difficult to replace him. The Board of Management especially wished to express their appreciation of the way in which he had met the great increase of work consequent upon the reception of very large numbers of wounded and sick soldiers in addition to the ordinary civilian patients.

War Increase and Expenditure for Thirteen Months.

The Chairman of the Finance Committee (Mr. Charles Larking) gave a brief résumé of the War Finance of the Institution, and explained that the result of the special appeal during thirteen months had resulted in the receipt of £10,500 from the county and city. Of this amount, £6,500 had been expended on the new buildings, tents, equipment and stores, necessary for the accommodation of the wounded men from the Front. The balance of £4,000 was given for the maintenance of beds under the scheme, whereby subscriptions of £5 per month had been sent from various workshops, households and individuals. A further sum of £9,400 had been received from the War Office on account of the soldier patients admitted, so that the total expenditure in connection with the wounded soldiers during the past thirteen months had been about £23,400. The hospital had also received a large amount of stores and goods, and much voluntary service. It was gratifying and praiseworthy that the peace-time subscriptions had been fully maintained. Under the £5 a month scheme, in addition to the £4,000, a sum of about £600 a month was still being received from this source. Had it not been for this fund, the Norfolk and Norwich Hospital could not have carried on its beneficent work during war-time.

Four and a Half Per Cent. War Loan *v.* Consols.

The Consols held by the hospital amounting to £12,713, yielding £320 a year, had been converted into 4½ per cent. War Loan Stock amounting to £8,417, yielding £379 per annum, or an additional income of £59 a year.

Rates of Subscription: Twelve months, 6/6; Six months, 4/-; Three months, 2/-, post free to any address in the United Kingdom. Abroad, Twelve months, 8/8; Six months, 5/-; Three months, 2/6, post free.—Address The Subscription Dept., "The Hospital," The Hospital Building, 28/29 Southampton Street, Strand, London, W.C.

